# Commentary: Visual Fixation in Human Newborns Correlates with Extensive White Matter Networks and Predicts Long-Term Neurocognitive Development

**DOI:** 10.3389/fnins.2016.00215

**Published:** 2016-05-18

**Authors:** Sarah H. Baum, Ryan A. Stevenson

**Affiliations:** ^1^Vanderbilt Brain Institute, Vanderbilt UniversityNashville, TN, USA; ^2^Department of Psychology, University of Western OntarioLondon, ON, Canada; ^3^Brain and Mind Institute, University of Western OntarioLondon, ON, Canada

**Keywords:** autism, visual perception, visual fixation, structural imaging, typical development

From early embryonic neural crest formation to postnatal synaptic pruning, each stage of development can strongly impact the structural and functional integrity of the mature nervous system. Changes in early development can have cascading downstream effects as more complex neurocognitive operations emerge. The visual system's maturation is highly dependent on early postnatal visual input and eye movements, both of which are directed by an individual's environment and where he or she looks within that environment. Despite the clear relationship between inputs to the visual system and healthy neurocognitive development, surprisingly little work has been reported on the influence of patterns of gaze and fixation on subsequent neurodevelopment.

To understand the early building blocks of visuocognitive development, Stjerna et al. collected and reported two sets of longitudinal data (Stjerna et al., 2015). The first cohort (*n* = 57) was initially assessed as newborns, when visual fixation (VF) and gaze behavior (GB) were measured. Furthermore, this group underwent a diffusion tensor imaging (DTI) scan series, providing a way to begin to connect behavioral findings to differences in the underlying neural pathways likely to support these functions. This group was later tested at 2 years of age with multiple subscales of the Griffiths Mental Developmental Series to assess eye-hand coordination, visual performance, and locomotor function.

Stjerna et al. found that infants who showed high performance in VF also showed better scores on the eye-hand coordination subscale at the 2 year follow up. Interestingly, the relationship between individual differences in visual skills at birth and at age 2 was also observable in DTI measures of structural connectivity. Specifically, there was a significant correlation between VF and white matter integrity in each large volume of interest examined covering the entire white matter skeleton.

As a validation of these findings, a second, larger cohort (*n* = 1410) was also assessed as newborns and was scored on VF. This group was later tested at 5 years of age and showed a significant positive relationship between VF during infancy and visual-motor, visual reasoning, and motor testing at the follow up.

This study is one of the first to empirically characterize the visual-cognitive cascade through development. The ability to maintain fixation on a moving object is a powerful index of the proficiency of the newborn visual system. Furthermore, this simple measure predicted performance in certain visual tasks at both 2 and 5 years of age, strongly suggesting that visual fixation is an important determinant for the developing visual system. Importantly, individual differences in behavior were also correlated with white matter integrity. Thus, these differences in the underlying neural network likely provide the necessary structural architecture to scaffold ongoing visuocognitive development.

In addition to their immediate relevance for the developing visual system, these findings also have particular importance for *atypical* visuocognitive development. Given these findings that the ability to simply maintain fixation on an object as an infant is predictive of later visuocognitive abilities, individuals who exhibit abnormal patterns of eye movement as an infant may show later weaknesses in visuocognitive abilities. This may in fact be the case in autism spectrum disorders (ASD), particularly in regards to the trajectory of visual exploration for simple and complex as well as non-social and social stimuli. For example, infants who are later diagnosed with ASD show a decline in fixation time on the eyes of a face stimulus during the interval from 2 to 6 months of age (Jones and Klin, 2013). Atypical gaze patterns in ASD have also been observed much later in development, including deficits in eye gaze sensitivity (Campbell et al., 2006), detecting a face with direct eye gaze (Senju et al., 2005), and complex facial expressions (Boraston et al., 2008). Many studies of individuals with ASD have also shown later developmental differences in network connectivity as measured using both DTI and functional MRI, including reduced connectivity in key nodes in the visuospatial network during the processing of both non-social (Damarla et al., 2010) and social stimuli (Kleinhans et al., 2008).

In light of previous research showing changes in connectivity later in development, the present behavioral and structural connectivity findings by Stjerna et al. in very early postnatal development support the combined use of simple visual behaviors and structural connectivity for potential use in identifying early biomarkers of ASD and predictors of later visual-cognitive functions in toddlers. Therefore, it would be informative to replicate these findings while including widely used behavioral screening tools such the Autism Quotient (which cannot be administered under 4 years of age) as well as measures of adaptive behavior like the Vineland Adaptive Behavioral Scales to determine if individual differences in VF at birth also predicted future social/cognitive traits at a later follow up. Longitudinal studies focusing on infant siblings of children with ASD would also be highly revealing of how early differences in lower level sensory and sensorimotor function can greatly impact later higher level sensory and cognitive function. Given that the development of speech and language skills relies on the integration of visual and auditory information (Foxe et al., 2015), additional assessments looking at early auditory and audiovisual integration skills could provide evidence for the hypothesis that ASD is the result of changes in the early development of low-level processes that initiate a divergent developmental cascade and ultimately result in the canonical difficulties that define ASD (Stevenson et al., 2014).

While ASD has long been studied from the perspective of higher-order cognitive differences (Baron-Cohen, 1989; Happé, 1999), recent focus has turned toward the concept that differences in sensory function may have cascading impacts, changing the developmental trajectories of the cognitive functions that are associated with ASD, including socio-communicative abilities. These findings have, in fact, led to changes in the diagnostic criteria for ASD, in which sensory disturbances are now included in the DSM-5. Stjerna et al. findings provide empirical evidence that patterns of eye movement may indeed impact downstream cognitive development, or may at the very least provide a measurement tool predictive of such downstream changes.

## Author contributions

All authors listed, have made substantial, direct, and intellectual contribution to the work, and approved it for publication.

## Funding

SB is supported by the Autism Speaks Meixner Postdoctoral Fellowship in Translational Research (#9717). RS is supported by a Banting Postdoctoral Fellowship granted by the Canadian Natural Sciences and Engineering Research Council (NSERC) and the Autism Research Training Program funded by The Canadian Institutes of Health Research (CIHR).

### Conflict of interest statement

The authors declare that the research was conducted in the absence of any commercial or financial relationships that could be construed as a potential conflict of interest.
